# Treatment of pulmonary arterial hypertension: A review of drugs available for advanced therapy

**DOI:** 10.7196/SARJ.2019.v25i1.236

**Published:** 2019-04-12

**Authors:** E Wilken, S Bennji, G Symons, P G Williams, B Allwood

**Affiliations:** 1 Division of Pulmonology, Department of Medicine, Tygerberg Hospital and University of Stellenbosch, Cape Town, South Africa; 2 Division of Pulmonology, Department of Medicine, University of Cape Town, South Africa; 3 Centre of Chest Disease and Critical Care, Milpark Hospital, Johannesburg, South Africa

**Keywords:** pulmonary hypertension, pulmonary arterial hypertension, drugs to treat pulmonary hypertension, guidelines to treat pulmonary arterial hypertension, review of pulmonary arterial hypertension

## Abstract

Pulmonary hypertension (PH) has traditionally been considered a rare disease with a uniformly poor prognosis. However, this was prior to the introduction of advanced therapies for this condition, and more recent registries in the treatment era have shown 5-year survival rates of up to 65%. Prior to 2000, there was only one licensed therapy for pulmonary arterial hypertension (PAH); less than 20 years later, the US Food and Drug Administration has approved 14 different medications for PAH. This review aims to summarise for the general pulmonologist the evidence for the current internationally available advanced therapies for PAH (World Health Organization Group I disease), which is characterised haemodynamically by the presence of precapillary PH in the absence of another cause. The benefit of these agents, either alone or in combinations, is now undisputed and their use is advocated in all current international guidelines for PAH. The improvement in survival of patients with PAH over the concurrent timeline emphasises the importance both of the availability and usage of effective therapies and of patients being seen in specialist centres, where physicians are familiar with using these therapies.

## Background

**Fig. 1:**
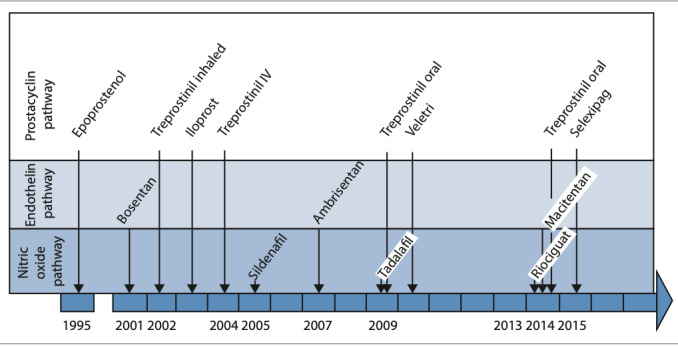
Approved therapies for pulmonary arterial hypertension. IV = intravenous

Pulmonary hypertension (PH) has traditionally been considered a rare disease with a uniformly poor prognosis. Indeed, in the 1990s, international registry data for primary PH (now called pulmonary arterial hypertension (PAH)) estimated the 5-year survival rate to be 34%.^[1]^ However, this was prior to the introduction of advanced therapies for this condition, and more recent registries in the treatment era have shown 5-year survival rates of up to 65%.^[2]^ Prior to 2000, there was only one licensed therapy for PAH; today, less than 20 years later, the US Food and Drug Administration (FDA) has approved 14 different medications for PAH (Fig. 1). These are used either alone or in combinations to improve symptoms and attain current survival rates.

PH is both a haemodynamic and a pathophysiological condition. It has been defined as an increase >25 mmHg in mean pulmonary arterial pressure (PAP) at rest, measured by right-heart catheterisation, since the first World Symposium on Pulmonary Hypertension in 1973.^[3]^ The causes are conventionally classified into five major groups (Table 1).^[4]^

**Table 1:** Clinical classification of pulmonary hypertension^[4]^

**Description**
1. PAH
1.1 Idiopathic PAH
1.2 Heritable PAH
1.3 Drugs and toxins induced
1.4 Associated with:
1.4.1 Connective tissue disease
1.4.2 HIV infection
1.4.3 Portal hypertension
1.4.4 Congenital heart disease
1.4.5 Schistosomiasis
1.5 PAH long-term responders to calcium channel blockers
1.6 PAH with overt features of venous/capillaries (PVOD/PCH) involvement
1.7 Persistent PH of the newborn syndrome
2. PH due to left-heart disease
2.1 PH due to heart failure with preserved ejection fraction
2.2 PH due to heart failure with reduced ejection fraction
2.3 Valvular heart disease
2.4 Congenital postcapillary obstructive lesions
3. PH due to lung diseases and/or hypoxia
3.1 Obstructive lung disease
3.2 Restrictive lung disease
3.3 Other lung disease with mixed restrictive/obstructive pattern
3.4 Hypoxia without lung disease
3.5 Developmental lung disorders
4. PH due to pulmonary artery obstruction
4.1 Chronic thromboembolic pulmonary hypertension
4.2 Other pulmonary artery obstructions
5. PH with unclear and/or multifactorial mechanisms
5.1 Haematologic disorders
5.2 Systemic disorders
5.3 Others
5.4 Complex congenital heart disease

PAH = pulmonary arterial hypertension

PVOD = pulmonary veno-occlusive disease

PCH = pulmonary capillary haemangiomatosis

PH = pulmonary hypertension

This review aims to summarise for the general pulmonologist the evidence that supports the current internationally available advanced therapies for PAH. The World Health Organization (WHO) classifies PAH as a Group I disease, which is characterised haemodynamically by the presence of precapillary PH in the absence of another cause. All other groups, except Group II, are characterised by precapillary PH with a defined cause. Group II, the most common cause of PH, is defined as being characterised by postcapillary PH, but in a proportion of patients there may be an additional precapillary component.

The definition of PH has recently been amended, and it is now recommended that precapillary PH be defined as a mean PAP >20 mmHg (previously >25 mmHg), with pulmonary artery wedge pressure (PAWP) ≤15 mmHg and a pulmonary vascular resistance (PVR) >3 Wood units.^[4]^ Group I PAH is a relatively small group, which is further divided into: idiopathic PAH; heritable PAH; PAH induced by drugs or toxins or PAH associated with collagen vascular disease, HIV infection, portal hypertension, congenital heart disease or schistosomiasis. Patients who have a long-term response to calcium channel blockers (CCBs) are recognised as a distinct group owing to their better long-term prognosis. Patients with overt features of venous and capillary involvement (previously termed pulmonary veno-occlusive disease) are recognised as having a significantly worse prognosis and are thus also grouped separately. Patients with portopulmonary hypertension and PAH associated with connective tissue disease have poorer long-term outcomes than patients with idiopathic PAH.^[5,6]^

The advanced therapies currently available target one of three metabolic pathways implicated in the pathogenesis of PAH, namely: the nitric oxide-cyclic guanosine monophosphate (NOcGMP) pathway; the endothelin pathway, and the prostacyclin pathway. High-dose CCBs can be used as first-line therapy in patients who exhibit vasoreactivity to nitric oxide (NO) at right-heart catheterisation; however, current recommendations support performing vasoreactivity testing only in patients with idiopathic PAH, heritable PAH and drug-induced PAH.^[7]^ Vasoreactivity testing performed in other PH classes can yield results that can be confusing and misleading, and in the presence of a raised pulmonary capillary wedge pressure (PCWP) may be dangerous. The use of CCBs at the very high doses recommended remains controversial, given that advanced therapies are now available, and extreme caution should be exhibited when managing cases with potential venous or capillary involvement.

## Nitric oxide – cyclic guanosine monophosphate enhancers

NO stimulates the conversion of guanosine triphosphate (GTP) to cGMP, which, in turn, activates protein kinases that specifically regulate ion channels and alteration of intracellular cyclic nucleotide concentrations. NO not only results in dilation of vascular smooth muscle of the arterial and venous vasculature but also has antiproliferative effects. The phosphodiesterase type 5 (PDE-5) enzyme is responsible for the degradation of cGMP and is found in substantial amounts in the pulmonary vasculature. Its expression is upregulated in PAH and leads to the increased metabolism of NO-derived cGMP. The consequent reduced levels of cGMP lead to altered calcium handling, vasoconstriction and smooth muscle cell proliferation. The inhibition of PDE-5, in turn, leads to vasodilation.^[8,9]^

### Sildenafil

Sildenafil is an orally active, selective inhibitor of phosphodiesterase type 5 (PDE-5i) and its effects peak after 60 minutes. Because of cost and availability, it is often used as the first-line agent in PAH in South Africa (SA). The use of sildenafil monotherapy in patients with PAH has shown beneficial results in three randomised control trials (RCTs) (http://www.ajtccm.org.za/public/docs/Table_2). ^[10,12]^ Galiè *et al*.^[10]^ conducted the largest of these trials, and compared sildenafil at different doses (20 mg, 40 mg and 80 mg three times a day) to a placebo in 277 patients over 12 weeks. Significant improvements were found in 6-minute walk distance (6MWD) (up to 50 m with the 80 mg dose), WHO functional class and haemodynamic parameters. Of note is that the improvement in 6MWD was retained one year later at follow-up. Flushing, diarrhoea and dyspepsia were the main side-effects. The smaller studies demonstrated similar findings (Appendix 1). The estimated minimal important difference in 6MWD for patients with PAH is 33 m.^[13]^

### Tadalafil

Tadalafil is a PDE-5i with a maximum effect after 75 - 90 minutes and, like sildenafil, has FDA approval for treatment of both PAH and erectile dysfunction. It has the advantage of being a once-daily oral preparation, with a similar side-effect profile to that of sildenafil. In the PHIRST study,^[14]^ 405 patients were treated with tadalafil at a range of doses (2.5 mg, 10 mg, 20 mg or 40 mg once a day) over 16 weeks and compared with a placebo group. Significant favourable results in exercise capacity (6MWD improvement of 33 m), haemodynamic parameters and time to clinical worsening were seen with the 40 mg dose. Lower doses did not appear to have the same significant effects. It is important to note that 53% of the subjects enrolled in this study received background bosentan therapy, and in these patients, lesser effects were seen than in treatment-naive patients. The known pharmacokinetic cytochrome P450 3A4 interaction between bosentan and both sildenafil and tadalafil may have accounted for this blunting of effect to some extent.^[14]^

### Vardenafil

Vardenafil is an oral PDE-5i that is administered twice daily. In an RCT, 66 treatment-naive PAH patients were treated with vardenafil for a total of 12 weeks (5 mg daily for the first 4 weeks and then escalated to twice daily). The effect was significant, with 6MWD – the primary outcome – increasing by 69 m. Favourable effects on both symptoms and haemodynamic parameters (i.e. cardiac index, mean PAP and pulmonary vascular pressure) were noted. The sideeffect profile was mild and transient, with headache and flushing being predominant.^[15]^

### Riociguat

Riociguat is not a PDE-5i, but stimulates soluble guanylate cyclase. It therefore also involves the NO-cGMP pathway, by increasing the conversion of GTP to cGMP. In the 12-week PATENT-1 trial, 443 patients were randomised to receive a placebo or riociguat in individually adjusted doses of up to 2.5 mg three times daily.^[16]^ Despite 50% of patients being treated with a background endothelin receptor antagonist (ERA) or a prostanoid, the use of riociguat resulted in a significantly improved 6MWD (36 m), regardless of background treatment. Riociguat also significantly and consistently improved the secondary endpoints of pulmonary haemodynamic parameters, WHO functional class and time to clinical worsening, and decreased *N*-terminal pro B-type natriuretic peptide (NT-proBNP) levels. The most common serious adverse event was syncope.^[16]^

In the PATENT-2 study, 396 patients were evaluated. Of these, 197 received riociguat monotherapy and 199 received riociguat combined with either an ERA or a prostacyclin. The improvements in 6MWD, WHO functional class and NT-proBNP levels were maintained at follow-up 2 years later. The survival rate at 2 years was 93%, and 79% of patients showed no clinical worsening. Serious adverse events were recorded in 238 patients (60%); 11% discontinued treatment because of an adverse event. Hypotension and syncope occurred in 13% and 10% of patients, respectively. This translates to 6.2 and 5.9 cases per 100 patient-years for hypertension and syncope, respectively.^[17]^

## Endothelin receptor antagonists

The endothelin system, and specifically endothelin-1 (ET-1) and endothelin receptor types A and B, is implicated in the pathogenesis of PAH. Raised ET-1 levels have been found in both plasma and lung tissue of PAH patients.^[18]^ ET-1 causes potent vasoconstriction and proliferation of smooth muscle and promotes vascular and interstitial remodelling by fibroblast activation, leading to proliferation of smooth muscles and endothelial cells.

### Bosentan

Bosentan is an oral antagonist of endothelin receptors A and B. It was the first oral therapy approved for the treatment of idiopathic PAH and PAH related to connective tissue disease. Four randomised trials^[19–22]^ have evaluated bosentan monotherapy (Study-351, BREATHE-1, BREATHE-5 and EARLY) and have found significant improvement in 6MWD (of up to 76 m), haemodynamics and time to clinical worsening with treatment (http://www.ajtccm.org.za/public/docs/Table_2).. The most notable side-effect associated with bosentan was an increase in liver enzymes in ~ 10% of patients. Although the elevated levels of hepatic aminotransferases appear to be dose dependent and reversible on cessation of therapy, close monitoring of liver function is necessary during therapy.

### Macitentan

Macitentan is a once-daily oral preparation that, like bosentan, inhibits endothelin receptor types A and B. In the SERAPHIN study, 742 patients were randomised to receive either macitentan (10 mg or 3 mg daily) or a placebo, for between 85 and 104 weeks.^[23]^ Importantly, almost two-thirds of patients were already receiving background therapy for PAH. The primary endpoint was the time from initiation of treatment to the first occurrence of a composite endpoint (death, atrial septostomy, lung transplantation, initiation of treatment with prostanoids or worsening of PAH), which occurred significantly less in the treatment groups: 31.4% in the 10 mg group and 38.0% in the 3 mg group v. 46.4% in the placebo group. Use of macitentan also significantly reduced the composite endpoint of mortality and hospitalisation due to PAH (21% in the 10 mg group, 26% in the 3 mg group and 34% in the placebo group) (p<0.001). This endpoint was driven largely by hospitalisations. The 6MWD at 6 months improved by 16.8 m in the 3 mg group and by 12.5 m in the 10 mg group, and both groups exhibited improvements in WHO functional class compared with the placebo group. Side-effects common to ERAs were found and included headaches, nasopharyngitis and anaemia; however, there was no increase in abnormal liver function.

### Ambrisentan

Ambrisentan, a once-daily oral inhibitor of endothelin receptor type A, has been studied in two large RCTs (ARIES 1 and 2) and demonstrated efficacy on symptoms, exercise capacity, haemodynamic parameters and time to clinical worsening. The ARIES 1 trial was performed over 12 weeks and included 202 patients. ARIES 2, which was an extension of the initial trial and included 192 patients, ran over 48 weeks.

Different doses of ambrisentan (5 mg or 10 mg in ARIES 1, and 2.5 mg or 5 mg in ARIES 2) were compared to a placebo. The 6MWD increased significantly in all treatment groups: by 31 m and 51 m for the 5 mg and 10 mg doses, respectively, in ARIES 1, and by 32 m and 59 m for the 2.5 mg and 5 mg doses, respectively, in ARIES 2. Improvements in time to clinical worsening, WHO functional class, Borg dyspnoea scores, SF-36 scores and NT-proBNP were also observed. Side-effects observed were similar to those for other ERAs and included oedema, sinusitis, nasal congestion, flushing, headache, constipation, abdominal pain and palpitations. The incidence of abnormal liver function ranged from 0.8% to 3%; however, transaminase levels did not exceed three times the normal range.^[24]^

## Prostacyclin pathway agonists

The prostacyclin agonists are a group of drugs that act via the prostaglandin I2 (PGI2) receptor to cause vasodilation and inhibit smooth muscle cell proliferation and platelet aggregation. Upon activation of the PGI2 receptor, adenosine triphosphate is converted to cyclic adenosine monophosphate, which, in turn, increases protein kinase A activity and so leads to relaxation of vascular smooth muscle cells.^[25,26]^

Prostacyclin agonists have been considered the gold standard of treatment for PAH Group I, and are recommended internationally as first-line treatment for patients classified as New York Heart Association (NYHA) functional class IV, and as an add-on treatment for patients classified as NYHA functional class III who are already on the maximal tolerable dose of an ERA and PDE-5i, or both.^[27,28]^ The prostacyclin pathway agonists include epoprostenol, Veletri®, treprostinil (intravenous, subcutaneous, oral or inhaled), inhaled and intravenous iloprost, and selexipag.

### Epoprostenol

Epoprostenol is a synthetic analogue of endogenous prostacyclin and is administered intravenously via a central venous line. The initial dose ranges from 1 to 12 ng/kg/minute, which can be up-titrated every week or two. There is no maximum dose, and the drug is titrated until a therapeutic response or dose-limiting toxicity is seen.^[29]^

The first RCT investigating the effects of epoprostenol was published in 1990^[30]^ and demonstrated that continuous intravenous prostacyclin produced a sustained reduction in PVR. In 10 patients treated for 2 months, six had reductions of >10 mmHg in mean PAP and a decrease of >30% in total PVR.

In 1996, a multicentre, open-label RCT compared the effects of continuous intravenous infusion in 81 patients with severe primary PH. Results showed that epoprostenol produced symptomatic improvement. After 12 weeks of therapy, the median change in 6MWD was an increase of 31 m from baseline in the treatment group, compared with a loss of 29 m in the conventional group. Functional class improved in 40% of subjects on therapy but in only 3% on conventional therapy. In addition, there was significant haemodynamic improvement with regard to both mean PAP and PVR. Strikingly, there were significant improvements with regard to mortality, as no deaths occurred in the treatment group.^[31]^ Epoprostenol is therefore the only monotherapy associated with beneficial mortality data, leading many experts to conclude that epoprostenol should be considered as the first-line treatment in patients with severe PAH (WHO functional class IV).

The most severe adverse effects associated with epoprostenol relate to the infusion system, including pump malfunction, thrombosis, interruption of the infusion and central venous catheter infection, which might increase morbidity and mortality. Other drug-related side-effects include flushing, dizziness, headache, fever, arthralgia, influenza-like symptoms and jaw pain.^[32]^

### Veletri (epoprostenol for injection)

Veletri is an epoprostenol formulation administered by continuous intravenous infusion. It offers increased stability relative to other available epoprostenol preparations. This has reduced the therapeutic burden usually associated with epoprostenol, as infusion pump cassettes can be prepared in advance and administration can be at room temperature, without the need for cooling with ice packs.^[33]^ It is considered safe and as effective as the other epoprostenol formulation, with the added advantage of improved storage conditions and patient convenience.^[34]^

### Treprostinil

Treprostinil is a tricyclic benzindene analogue of prostacyclin, with similar antiplatelet and vasodilatory actions, including acute pulmonary vasodilation. It can be administered subcutaneously, intravenously, orally or by inhalation. The subcutaneous injection is not used often owing to severe pain experienced at the injection site. The initial dose of intravenous treprostinil is 1.25 ng/kg/minute, which can be titrated up every week. The initial dose for inhaled administration is 18 μg per treatment and can also be titrated up. The initial oral dose is 0.25 mg every 12 hours.^[35]^

The first prospective evaluation of intravenous treprostinil demonstrated that it improved exercise capacity at 12 weeks, based on an increase of 82 m in 6MWD and an increase of 146 s in NaughtonBalke treadmill time. Similarly, Borg dyspnoea scores and WHO functional class improved. Haemodynamic parameters improved significantly: mean PAP decreased by 9%, cardiac index increased by 29% and PVR decreased by 33% compared with baseline assessments. The most frequent side-effects were those commonly attributed to and expected in prostacyclin therapy.

Intravenous treprostinil has a number of potential advantages over intravenous epoprostenol. These include a longer half-life, which could reduce life-threatening crises in the event of sudden infusion interruption, and stability at room temperature, which renders ice packs unnecessary and makes it more convenient to the user. Finally, intravenous treprostinil can be prepared every 48 hours rather than every 24 hours, as is required with epoprostenol.^[36]^

Oral treprostinil can be used as initial therapy in patients with less severe PAH (class II and III symptoms), as was demonstrated in a randomised double-blind, placebo-controlled study.^[37]^ Improvements in 6MWD were seen at 12 weeks in both the intent-to-treat (ITT) and modified ITT population (26 m and 23 m, respectively). The average dose of oral treprostinil achieved by modified ITT patients who completed the assessments at 12 weeks was 3.4 mg. The most common adverse effects were headache, nausea, diarrhoea, jaw pain and vomiting, similar to effects seen in other prostacyclin therapies. Oral treprostinil has also been approved for use as an add-on therapy for PAH.^[38]^

Inhalational treprostinil was evaluated in a 12-week randomised double-blind, placebo-controlled trial of 235 patients, as an add-on therapy to either bosentan or sildenafil, and was shown to improve 6MWD significantly (+20 m).^[39]^

### Iloprost

Iloprost is a prostacyclin analogue that is administered by inhalation or by continuous intravenous infusion. A randomised placebo-controlled trial of 203 patients demonstrated that long-term inhalation of aerosolised iloprost as add-on therapy improved exercise capacity: 6MWD increased by 58.8 m and both NYHA functional class and haemodynamic parameters improved.^[40]^ The main disadvantage of inhaled therapy is that frequent administration is required (between six and nine times per day). Other side-effects are similar to that of prostacyclin. The major concern regarding the intravenous route is the lack of robust data showing outcome benefit equivalent to that of epoprostenol while relying on short-term data showing equivalent haemodynamic efficacy. It remains the only parenteral prostanoid available in some countries (e.g. Germany and New Zealand). Iloprost is cheaper than other agents in its class and its longer intravascular half-life makes it an attractive option for use in the SA setting.

### Selexipag

Selexipag is an oral prostacyclin receptor with high selectivity for the PGI2 receptor, which causes vasodilation of the pulmonary circulation. The starting dose is 400 µg and is individually uptitrated until side-effects are seen.^[41]^ The GRIPHON trial,^[42]^ a large multicentre, double-blind, randomised placebo-controlled study, showed that selexipag was associated with a reduction in a composite end of death or PAH-related complications (27% in the treatment group v. 42% in the placebo group), largely driven by hospitalisations and disease progression. In that study, selexipag was used as an add-on therapy in ~ 80% of patients who received a stable dose of an ERA, a PDE-5i, or both. Most adverse effects were similar to known sideeffects of other prostacyclin therapies.

The approved therapies targeting the prostacyclin pathway can provide patients with significant additional benefit. However, administration can be complicated, with up-titration needing to be carefully balanced against side-effects. Because of the potential for a number of serious pitfalls in using these agents, it is recommended that these therapies should be prescribed only by healthcare professionals experienced and comfortable in their use.^[43]^

## Combination therapy

The combined use of agents that target different metabolic pathways is a promising therapeutic option for PAH, and works by either targeting the different pathways simultaneously or adding a synergistic benefit. Combination therapy is now recommended as add-on in PAH patients who exhibit a suboptimal response to monotherapy (sequential drug combination therapy), and many patients may benefit from the addition of a third agent if dual therapy is unsuccessful. There is also increasing evidence for the use of combination therapy at treatment initiation, with the aim of targeting multiple pathogenic pathways from the outset and so limiting vascular remodelling.

Most studies evaluating combination therapy have assessed whether targeting two pathogenic pathways at once is superior to monotherapy, and two recent meta-analyses confirmed that combination therapy confers an estimated reduction of 35% in the relative risk of clinical worsening.^[27,44,45]^

It should be stressed that with the improvement in PH survival, clinical trial design has evolved in recent years. Studies have moved away from investigating single-parameter, short-term endpoints, with the focus now on more clinically meaningful endpoints over longer periods and new agents often being added to background therapies. The strongest evidence for the use of combination therapy can probably be inferred from the large-scale SERAPHIN and GRIPHON studies. These trials both used a similar composite and clinically relevant ‘time to clinical worsening’ endpoint, comprising mortality, need for additional therapy or markers of clinical worsening such as admission to hospital or worsening functional class. In both these studies, trial medication was added to baseline therapy if already initiated. The benefit of the investigated therapy (namely macitentan in the SERAPHIN trial and selexipag in the GRIPHON trial) was maintained and was, in fact, additive to the therapeutic effect of baseline therapy in both trials, thereby approximating clinical practice better than single-parameter outcome trials.

A recent event-driven trial also used a clinical composite endpoint to show the clear benefit of initial combination therapy with tadalafil and ambrisentan.^[46]^ Dual combination therapy, either *de novo* or sequential, is now commonly used in clinical practice and is recommended by most guidelines.^[27,47]^

### Combination of endothelin receptor antagonists and phosphodiesterase type 5 inhibitors

Evidence of the beneficial effect of combining bosentan and sildenafil was observed in the EARLY study, where 16% of patients who were on background sildenafil and then given bosentan showed significant haemodynamic improvement and clinical deterioration was prevented.^[21]^ In the COMPASS-2 study, McLaughlin *et al*.^[48]^ found that in 334 patients, the addition of bosentan to sildenafil did not improve time to a morbidity or mortality event, but 6MWD improved by 22 m at 16 weeks. However, this finding was considered exploratory. No other endpoints showed significant differences. Despite the lack of benefit, no new safety concerns were raised. A number of difficulties with this study were noted by the authors and definitive conclusions were therefore limited.

The addition of tadalafil to ERAs has been studied in a number of trials. As noted previously, the PHIRST study demonstrated additional benefit in adding tadalafil to subjects on background bosentan therapy.^[14]^ The addition of tadalafil to background ambrisentan was explored in two studies. Zhuang *et al*.^[49]^ were unable to draw definitive conclusions, but their results showed significant improvement in 6MWD and lesser clinical worsening, without additional adverse events. In the larger AMBITION trial, ambrisentan and tadalafil were tested alone and in combination in 500 treatment-naive participants.^[46]^ This double-blind study randomised participants to a combination of ambrisentan (10 mg) and tadalafil (40 mg) (n=253), ambrisentan (10 mg) plus a placebo (n=126), or tadalafil (40 mg) plus a placebo (n=121) for 24 weeks. Significantly fewer occurrences of the endpoints of clinical failure (death, hospitalisation, disease progression, unsatisfactory long-term response) were found in the case of combination therapy (18%) than in either of the monotherapy groups (34% and 28%, respectively). Secondary endpoints, including mean change in NT-proBNP and 6MWD, were also significantly better with combination therapy. The 6MWD improved by an average of 49 m in the case of combination therapy, compared with an improvement of 24 m in the monotherapy groups. However, more adverse events were found in the combination therapy group.

In the ATHENA-1 study, conducted in PAH patients who exhibited a suboptimal therapeutic response, the addition of ambrisentan was associated with haemodynamic, functional and biomarker improvement.^[50]^ The primary endpoint (change in PVR) was statistically significant (–33% from baseline) and the side-effect profile was the same as for ambrisentan, with a 10% discontinuation rate. Of note is that both sildenafil and tadalafil were used in the PDE-5i group.^[50]^

### Combination of prostaglandin analogues and oral agents

In the PACES-1 study, 267 patients with idiopathic PAH or associated PAH (due to connective tissue disease, shunts or use of an anorexigen) and who were on intravenous epoprostenol therapy, were given sildenafil or a placebo. Significant improvement in 6MWD and haemodynamic parameters and a delay to clinical worsening were observed in the sildenafil group. No benefit was seen in the Borg dyspnoea scores. Side-effects included headache and dyspepsia.^[51]^ PACES-2, the open-label extension over more than 3 years, captured longer-term survival (66% patients alive) and demonstrated sustained improvement in 6MWD.^[52]^ There was a clear benefit in dual therapy and the addition of sildenafil to background intravenous epoprostenol therapy appeared to be well tolerated.

Inhaled treprostinil was added to oral therapy in the TRIUMPH 1 study,^[39]^ an RCT of 235 patients with idiopathic or associated PAH. The primary endpoint (6MWD) improved significantly. Improvements were also seen in quality of life and biomarkers, but not in other secondary endpoints, including time to clinical worsening, Borg dyspnoea scores, functional class and symptoms. Patients on background bosentan (70%) had greater improvements than those on sildenafil (30%), although the authors conceded that the study was not designed or powered to draw definitive conclusions on combination superiority. The combination therapy appeared to be safe and well tolerated.

First-line combination therapy was tested in the BREATHE-2 trial, with the addition of bosentan to epoprostenol in 33 patients.^[53]^ Statistically non-significant trends towards improvement in all haemodynamic variables were seen compared with epoprostenol monotherapy.^[53]^ No significant differences in 6MWD or functional class were observed; however, the study was not powered for these endpoints and because the sample size was small, this finding did not allow a definitive conclusion. In a small open-label study called STEP,^[54]^ inhaled iloprost was investigated as add-on to stable bosentan monotherapy. The combination resulted in significant improvement in 6MWD and functional class and delayed clinical worsening, but no change in Borg dyspnoea scores. Combination therapy was well tolerated.

In the COMBI study, using iloprost with bosentan showed no additional benefit and there was no difference in any endpoints.^[55]^

### Combination of two phosphodiesterase type 5 inhibitors

Both riociguat and sildenafil work on the NO-cGMP pathway, and because of potential safety and efficacy concerns, the combination of these two drugs in PAH patients was explored in the PATENT PLUS study.^[56]^ Patients receiving sildenafil (20 mg three times a day) were randomised to a placebo or riociguat (up to 2.5 mg three times a day) for 12 weeks. Although blood pressure did not change in the initial study, the long-term extension study showed high rates of discontinuation due to hypotension and three deaths (reported as unrelated). The study was terminated by the investigators and sponsors, and because of potentially unfavourable safety signals with no evidence of positive benefit, the concomitant use of riociguat and a PDE-5i can now be considered contraindicated and is thus not advised.^[56]^

### Triple therapy

Although none of the RCTs reviewed was designed specifically to assess triple therapy, 33% - 45% of patients were on background combination therapy in the FREEDOM-C and GRIPHON studies and the study by Simonneau *et al*.^[38,42,57,58]^

## Drugs that are available in South Africa

In SA, sildenafil citrate (Revatio) and ambrisentan (Volibris) are registered for the treatment of PAH. Off-label use of tadalafil (Cialis) has been combined with ambrisentan as first-line therapy in some centres. Use of bosentan, iloprost and macitentan can be applied for with a Section 21 form to the South African Health Products Regulatory Authority.

## Conclusion

This review highlights in some detail the advances in PAH therapy and comments on the benefit of these agents, whether alone or in combinations. In the last 20 years there has been a considerable increase in the number of therapies approved for PAH. Unfortunately, the majority of these agents are not available in SA, for reasons that are not totally clear but are probably multifactorial, ranging from cost to lack of awareness and lack of advocacy from patients and doctors alike. However, the benefit of these agents, either alone or in combinations, is undisputed and their use is advocated in all current international guidelines for treating PAH. The improvement in survival of patients with PAH over the concurrent timeline emphasises the importance both of the availability and usage of effective therapies and of patients being seen in specialist centres, where physicians are familiar with using these therapies.

## Supplementary Tables

Appendix 1
Table 2: Summary of trials in pulmonary arterial hypertension (PAH)
