# Supplementary material for: Treatment of pulmonary arterial hypertension: A review of drugs available for advanced therapy
Source: Afr J Thorac Crit Care Med. 2019 Apr 12;25(1):10.7196/SARJ.2019.v25i1.236. doi: 10.7196/SARJ.2019.v25i1.236 (PMC8279001; doi:10.7196/SARJ.2019.v25i1.236)
Supplement: Supplementary file 1 — Appendix 1 Table 2: Summary of trials in pulmonary arterial hypertension (PAH) [file AJTCCM-25-1-236-S001.pdf]

Table 2: Summary of trials in pulmonary arterial hypertension (PAH):

Name / author	Patients n	Study drug	Control	Primary end-point(s)	Secondary end-point(s)	Findings	Side Effect
NO-cGMP enhancers							
Bharani	9	*Sildenafil*	Placebo	6MWD	Haemodynamic and symptom scores	Significant improvement in 6MWD (96 m), improvement in symptoms and haemodynamic parameters	None reported
Sastry	22	*Sildenafil*	Placebo	Exercise testing	Haemodynamic and QOL	Significant improvement in exercise treadmill time (211 s), CI and QOL. No change in PASP	None reported
SUPER	277	*Sildenafil*	Placebo	6MWD	Haemodynamic and symptom scores	Significant improvement in 6MWD (50 m in 80 mg group) and mPAP, no change in any other parameters<br>NOTE: more hospitalizations	Flushing, diarrhoea, dyspepsia. 4 deaths.
Zing	66	*Vardenafil*	Placebo	6MWD	Haemodynamic	Significant improvement in 6MWD (69 m) and haemodynamic	Mild and transient. Headache and flushing
PATENT	443 (PATENT-1)<br>*Background: ERA 44 % / prostanoids 6 %*<br>396 (PATENT-2)<br>18 (PATENT PLUS)<br>*Background: Sildenafil*	*Riociguat*	Placebo	6MWD<br>SBP	Haemodynamic and symptom scores<br>Haemodynamic and safety	6MWD improved (36 m; regardless of background treatment), improvement of haemodynamic, FC, time to worsening less<br>Long-term extension: 6MWD improved (53 m)<br>No change is SBP. No favourable effects	Headache, dyspepsia and oedema, hypotension<br>Hypotension, death (18 %)<br>Contra-indicated

<u>Endothelin receptor antagonists</u>							
Study-351	32	*Bosentan*	Placebo	6MWD	Haemodynamic, symptom scores	Improvement in 6MWD (67 m), PVR decreased, FC and symptoms improved.	Transient
BREATH-1	213	*Bosentan*	Placebo	6MWD	Symptom scores	Improvement of 6MWD (35 m in 250 mg), symptoms and FC	Dose related liver injury
BREATH-5	54	*Bosentan*	Placebo	Oxygen saturation (SpO2)	Haemodynamic and 6MWD	Effect on SpO2 was similar in both groups. PVR index reduced (-5.5 dyne.s.cm-5). 6MWD improved	Peripheral oedema, headache and palpitations
ARIES	202 (ARIES 1)<br>192 (ARIES 2)	*Ambrisentan*	Placebo	6MWD	Symptom scores	6MWD improved significantly in both ARIES 1 (31 m for 5 mg and 51 m for 10 mg) and ARIES 2 (32 m for 2.5 mg and 59 m for 5 mg), time to clinical worsening, WHO FC, BORG, SF-36 and BNP improved	Oedema, sinusitis, nasal congestion, flushing, headaches, constipation, abdominal pain and palpitations
<u>Prostacyclin pathway agonists</u>							
Rubin	24	*Epoprostenol*	Conventional therapy	PVR	Haemodynamic	A decrease of greater than 30% in total pulmonary vascular resistance (-7.9 Wood U). Sustained haemodynamic and symptomatic responses	Complications from drug delivery system

Barst	81	*Epoprostenol*	Conventional therapy	Survival and 6MWD	Haemodynamic	Improvement in 6MWD (31 m) and survival	Complications from drug delivery system
Tapson	16	*Treprostinil (IVI)*	Open label	6MWD	Haemodynamic and symptom scores	6MWD increased (82 m). Improvement in symptoms, FC and haemodynamic	Headache and cough
FREEDOM-M	350	*Treprostinil (oral)*	Placebo	6MWD	Symptom scores	6MWD increased (23 m), no change in FC, dyspnoea scores or clinical worsening	Headache, nausea, diarrhea, jaw pain and flushing
Olschewski	203	*Iloprost*	Placebo	6MWD and symptom scores	Haemodynamic	6MWD increased (36.4 m), hemodynamic, FC, symptom scores all improved significantly. Combined clinical endpoints was met in 16.8 % vs 4.9 % of patients	Flushing, jaw pain and syncope
Sitbon	1156	*Selexipag*	Placebo	Mortality and morbidity	6MWD and symptom scores	Primary endpoint event occurred in 27 % in selexipag group and 41.6 % placebo group (HR 0.6). Disease progression and hospitalization accounted for 81.9 % of events. 6MWD improved, but there was no difference in the other parameters	Headache, diarrhea, nausea and jaw pain
<u>Combination therapy</u>							
PACES	267	*Sildenafil*	Placebo	6MWD	Symptom scores	Significant improvement in 6MWD, haemodynamic and time to clinical worsening better. No benefit in Borg dyspnoea score. Benefit in dual therapy.	Headache and dyspepsia

	Background: epoprostenol						
PHIRST	405<br><br>Background: bosentan 53%	*Tadalafil*	Placebo	6MWD	Haemodynamic and symptom scores	Dose-response, only 40 mg dose met significant improvement (6MWD improved by 33 m), haemodynamics and time to clinical worsening. Not in lower doses.	Headache, myalgia and flushing
Zhuang	124<br><br>Background: ambrisentan	*Tadalafil*	Placebo	6MWD	Haemodynamic, FC, clinical worsening	Statistical difference in 6MWD (36 m), none in secondary end points	No differences in adverse events between groups
EARLY	185<br><br>Background: sildenafil 16 %	*Bosentan*	Placebo	6MWD and hemodynamic	Symptom scores	PVR improved significantly (-197 dyne.s.cm-5) (similar effect with or without sildenafil). No improvement in 6MWD or symptoms. Delay in clinical worsening and improvement in biomarker	Similar between groups: bosentan nasopharyngitis and liver abnormality
BREATH-2	33<br><br>Epiprostenol for 16 weeks	*Bosentan + Epoporstenol*	Placebo + Epoprostenol	PVR	Haemodynamic and clinical failure	PVR decreased but not significantly (-188 dyne.s.cm-5). Hemodynamic, 6MWD, dyspnoea fatigue rating, NYHA FC improved in both groups. No benefit	Leg oedema in bosentan group
COMPASS-2	334<br><br>Background: sildenafil	*Bosentan*	Placebo	Complex of morbidity and mortality	6MWD, symptom scores	No difference in morbidity or mortality. 6MWD improved, but no other endpoints were met	No new safety signals were observed
SERAPHIN	742<br><br>Background: PDE-5i 32 %, prostanoid 5 %	*Macitentan*	Placebo	Complex of morbidity and mortality	Morbidity and mortality<br><br>Symptom scores	Primary endpoint occurred in 31.4 % (10 mg), 28 % (3 mg) and 46.4 % (placebo). Worsening of PAH was the most frequent primary endpoint. Lower rates of hospitalization in macitentan group. 6MWD and FC also improved	Headache, nasopharyngitis, anaemia.
AMBITION	500	*Ambrisentan and tadalafil*	Ambrisentan or tadalafil	Complex of clinical failure	NT-proBNP	Primary endpoint occurred in 28 % (tadalafil monotherapy), 34 % (ambrisentan monotherapy) and 18 % (combination	More adverse events in combination (oedema,

					6MWD, WHO FC,	therapy). Significant difference in all endpoints. Dual therapy has benefit mostly driven by hospitalization	headache, nasal congestion, anaemia)
ATHENA-1	33<br><br>Background therapy: sildenafil / tadalafil	*Ambrisentan*	Placebo	PVR	Other haemodynamic parameters, functional parameters and biomarkers	Statistical significant improvement in PVR (-32%) and all secondary endpoints were met	Similar to ambrisentan but 10% of patients discontinued due to it
GRIPHON	1156<br><br>Background: ERA (15%), PDE-5i (32%), both (33%)	*Selexipag*	Placebo	Complex of morbidity / mortality	6MWD, WHO FC and exploratory end points	40 % reduction in first event of death or complication related to PAH. Significant improvement in 6MWD. No change in FC	Headache, diarrhea, and nausea. hyperthyroidism
Simonneau	43<br><br>Background: bosentan and/ sildenafil	*Selexipag*	Placebo	PVR	6MWD and symptom scores	30.3 % reduction in PVR. Improvement in haemodynamics. No difference in 6MWD, Borg dyspnea score and NT-proBNP	Headache, jaw pain, nausea, nasopharyngitis
COMBI	40<br><br>Background: bosentan	*Iloprost*	None	6MWD	FC, QOL	No significant effect on 6MWD (19 m), WHO FC or time to clinical worsening<br><br>No benefit observed	Pneumonia and coughing
STEP	67<br><br>Backround: bosentan	*Iloprost*	Placebo	6MWD	Efficacy endpoints	Significant improvement in 6MWD (26 m), FC and time to clinical worsening less. No change in Borg. Benefit in dual therapy	Well tolerated

FREEDOM	350 (FREEDOM-C)<br><br>Background: ERA 30 %, PDE-5i 25 %, ERA + PDE-5i 45 %<br><br>310 (FREEDOM-C2)<br><br>Background: ERA 17 %, PDE-5i 40 %, ERA + PDE-5i 40 %	*Treprostinil*	Placebo	6MWD	Clinical failure	Non-significant treatment effect in 6MWD (11 m), significant improvement in dyspnea fatigue index and combined 6MWD and dyspnea score, no change in time to clinical worsening, WHO FC and dyspnea score. No difference in any endpoints were observed in FREEDOM C2	Headache, nausea, diarrhea, vomiting and pain
TRIUMPH	235<br><br>Background: bosentan 70 %, sildenafil 30 %	*Treprostinil*	Placebo	6MWD	Clinical failure	Significant improvement in 6MWD (20 m). Improvement in QOL, NT-proBNP, no change I time to clinical worsening, Borg dyspnea score, WHO FC and symptoms. No evidence of benefit	Cough (54 %), headache, nausea, dizziness and flushing
6MWD (six-minute walking distance), QOL (quality of life), PASP (pulmonary artery systolic pressure), PVR (peripheral vascular resistance), FC (functional class), mPAP (mean pulmonary artery pressure), CI (cardiac index), SBP (systolic blood pressure), SpO2 (peripheral capillary saturation)							
